# Soft-Template Synthesis of Mesoporous Anatase TiO_2_ Nanospheres and Its Enhanced Photoactivity

**DOI:** 10.3390/molecules22111943

**Published:** 2017-11-10

**Authors:** Xiaojia Li, Mingming Zou, Yang Wang

**Affiliations:** 1 School of Fundamental Sciences, China Medical University, Shenyang 110122, China; xiaojia9784@126.com; 2 Dalian National Laboratory for Clean Energy, Dalian Institute of Chemical Physics, Chinese Academy of Sciences, Dalian 116023, China; mmzou@dicp.ac.cn

**Keywords:** mesoporous anatase TiO_2_, high surface area, photocatalytic

## Abstract

Highly crystalline mesoporous anatase TiO_2_ nanospheres with high surface area (higher than P25 and anatase TiO_2_) are prepared by a soft-template method. Despite the high specific surface area, these samples have three times lower equilibrium adsorption (<2%) than Degussa P25. The rate constant of the mesoporous anatase TiO_2_ (0.024 min^−1^) reported here is 364% higher than that of P25 (0.0066 min^−1^), for the same catalytic loading. The results of oxidation-extraction photometry using several reactive oxygen species (ROS) scavengers indicated that mesoporous anatase TiO_2_ generates more ROS than P25 under UV-light irradiation. This significant improvement in the photocatalytic performance of mesoporous spherical TiO_2_ arises from the following synergistic effects in the reported sample: (i) high surface area; (ii) improved crystallinity; (iii) narrow pore wall thicknesses (ensuring the rapid migration of photogenerated carriers to the surface of the material); and (iv) greater ROS generation under UV-light.

## 1. Introduction

The irradiation of a semiconductor with light of energy greater than its band gap (ΔEg) results in the formation of photogenerated holes and electrons, which are capable of oxidizing or reducing (directly or indirectly) nearby species. Such a scheme is frequently utilized for the transformation of pollutants (e.g., the oxidation of toxic organics, the reduction of metal ions) into less dangerous species (e.g., carbon dioxide, metal deposits). This process, called photocatalysis, is aided by the use of a semiconductor with an appropriate band gap, high specific surface area, optimum electron and hole transfer properties, and surface stability [1].

Owing to its appropriate surface stability, long carrier life time and mobility, solution phase dispersibility, and high photo corrosion resistance [2], titanium dioxide has been a semiconductor of choice. The morphology of the TiO_2_ photocatalyst [3,4,5] has proven to be an important factor for enhancing its catalytic activity. Thus, a variety of controlled synthesis methods have been attempted to synthesize well-defined particles with varied morphologies, such as nanoparticles, porous materials, etc. [6,7]. TiO_2_ with different morphologies and sizes have been fabricated with the purpose of further enhancing their photocatalytic activity [8,9]. Notably, TiO_2_ nanoparticles have been proven to be more effective than their bulk samples, mainly because their large surface area can provide more active sites [10,11]. On the other hand, high crystallinity is essential since it reduces the recombination of the photogenerated carriers. Hence, increasing the surface area and improving the crystallinity aids in improving the photoactivity of TiO_2_ [12,13,14].

In this paper, we report a simple method by using a soft-template to make well-defined highly crystalline mesoporous spherical TiO_2_. We use the TiO_2_ thus obtained to conduct the adsorption and photocatalytic degradation of organic dye. For comparison, we use commercial P25 TiO_2_ and anatase TiO_2_ as the reference photocatalysts, in keeping with standard practices reported so far [15]. The model pollutant used is methylene blue (MB). It may be noted that a high concentration of MB can cause hemolytic anemia, nausea, fever, and hypotension, etc. [16]. So, the treatment of pollutants containing MB is of utmost important. TiO_2_ could produce reactive oxygen species (ROS) after irradiation by UV-light. Moreover, ROS are the main influencing factor in the photocatalytic degradation of organic dye. In order to examine the generation of ROS, the method of Oxidation-Extraction Spectrometry (OES) for detecting ROS was taken here [17].

## 2. Results and Discussion

### 2.1. Formation Mechanism of the Mesoporous Spherical TiO_2_

**Figure 1:**
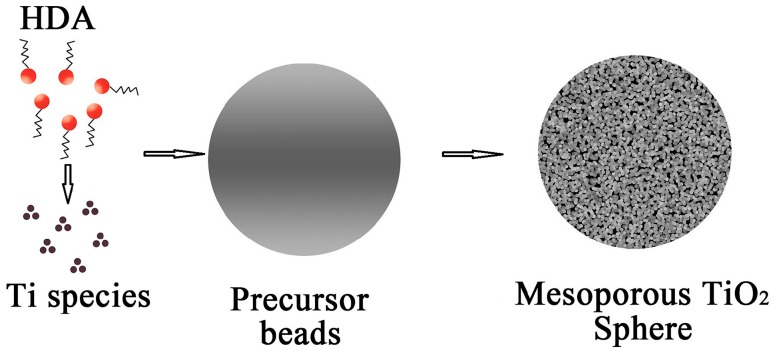
Schematic representation of the formation mechanism of the mesoporous spherical TiO_2_.

As shown in Figure 1, the mesostructures and monodisperse precursor beads were formed through a cooperative assembly process involving long-chain alkylamine and Ti(OCH(CH_3_)_2_)_4-*x*_(OH)*_x_* species/oligomers [18]. The resultant Ti(OCH(CH_3_)_2_)_4-*x*_(OH)*_x_* species on the hydrolysis of Titanium (IV) isopropoxide (TIP) participate in hydrogen-bonding interactions with amino groups of the Hexadecylamine (HDA). Such hybrid composites contain hydrophobic long-chain alkyl groups [19]. Meanwhile, further hydrolysis and condensation of the titanium species associated with the hybrid micelles results in the formation of a new liquid condensed phase rich in HDA and titanium oligomers. As the titanium oligomers polymerize further, the condensed phase becomes denser with time, and the formation of a mesostructured inorganic framework finally precipitates from the solvent. During hydrothermal treatment, amorphous TiO_2_ is known to experience a phase change to anatase via a dissolution and reprecipitation process, wherein dissolved titanate species rapidly nucleate to form nanocrystalline structures due to the high reactivity of the hydrothermal system. Further annealing in air induces the decomposition of HDA molecules and promotes the formation of crystalline mesoporous TiO_2_ spheres.

### 2.2. XRD of Mesoporous Spherical TiO_2_ and P25 TiO_2_

**Figure 2:**
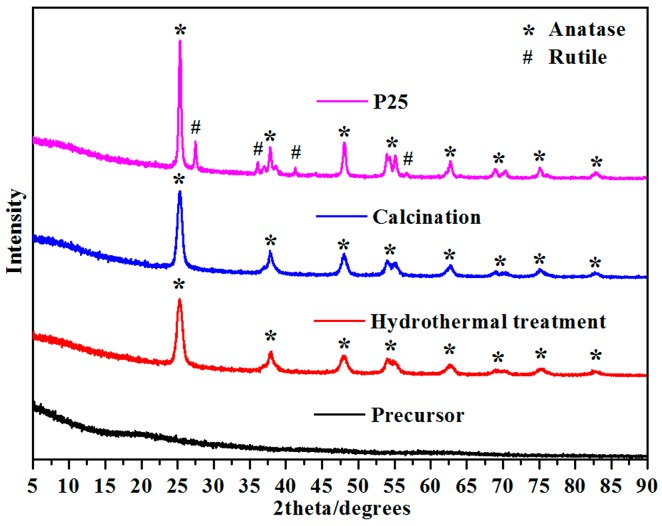
PXRD patterns of the precursor bead, mesoporous spherical TiO_2_ after hydrothermal treatment, mesoporous spherical TiO_2_ after calcination, and P25 TiO_2_.

**Figure 3:**
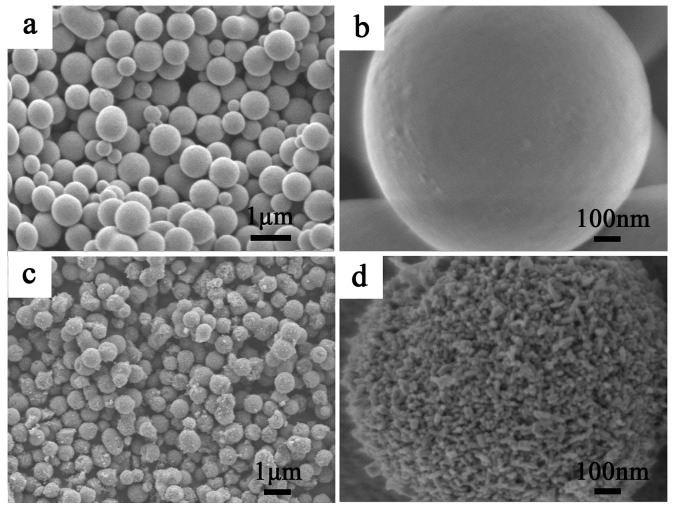
SEM of precursor beads (**a**,**b**) and mesoporous spherical TiO_2_ obtained after hydrothermal and calcination treatment (**c**,**d**).

Figure 2 shows the X-ray diffraction (XRD) patterns for the TiO_2_ nanospheres formed from precursors when hydrothermal treatment and product calcination is conducted at 500 °C for 2 h. This figure confirms the phase purity of the products at each stage. The TiO_2_ crystallizes in the space group I41/amd with refined lattice parameters of a = 3.7842 (2) Å and b = 9.5146 (2) Å by using the General Structure Analysis System (GSAS) package, as shown in Figure S1. Both TiO_2_ samples show relatively broad diffraction peaks due to the small crystalline domain sizes. The PXRD refinement of the product obtained shows that the average crystalline domain size is 46 nm, which agrees well with the SEM results shown in Figure 3d. On the other hand, for the P25 sample, the PXRD pattern shows a mixture phase of anatase and rutile TiO_2_, in agreement with results reported in the literature [20].

### 2.3. SEM of Mesoporous Spherical TiO_2_ and P25 TiO_2_

Figure 3 shows scanning electron microscopy (SEM) images of the calcined mesoporous spherical TiO_2_ prepared after the hydrothermal process. The precursor material (sample in Figure 3a) contained monodisperse beads with a diameter of 0.80 ± 0.05 μm. These beads possess very smooth surfaces without obvious granular features (Figure 3b). After hydrothermal treatment in a mixture of 40 mL ethanol and 20 mL deionized water, followed by calcination at 500 °C for 2 h, monodisperse TiO_2_ beads with a diameter of 0.60 ± 0.05 μm and comparatively rough surfaces were produced (Figure 3c), indicating a shrinkage of ≈25% in bead diameter during calcinations, owing to the removal of the template [21]. As illustrated by the high magnification SEM image (Figure 3d), these TiO_2_ beads contain nanocrystals, and pores are observed over the surface of the beads. By contrast, the P25 particles (Figure S2a,b) are present in the form of aggregated clusters without fixed morphology, and have a much larger size.

### 2.4. BET of Mesoporous Spherical TiO_2_ and P25 TiO_2_

**Figure 4:**
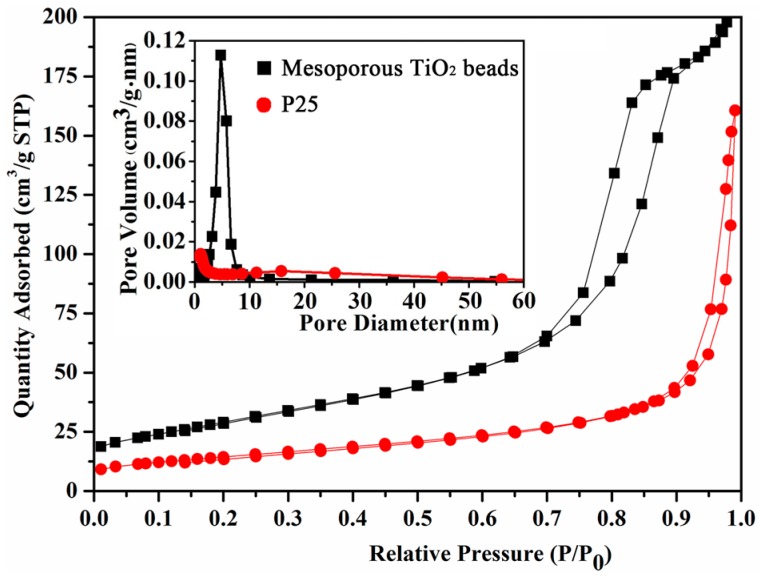
Nitrogen sorption isotherms of the mesoporous spherical TiO_2_ and P25 TiO_2_.

The textural properties of mesoporous spherical TiO_2_ and P25 TiO_2_ were analyzed by N_2_ adsorption. As shown in Figure 4, after the hydrothermal and calcination treatment, type IV isotherms with a sharp capillary condensation step at high relative pressures (P/Po ≈ 0.7–0.9) and H1 type hysteresis loops were observed for the mesoporous spherical TiO_2_. This indicates the relatively large pore sizes and uniform pore size distribution of this sample. The mesoporous spherical TiO_2_ had a specific surface area of 106.9 m^2^·g^−1^ and a narrow pore size distribution centered at 5.0 nm. For the P25 TiO_2_, the surface area was 52.1 m^2^·g^−1^, almost two times lower than that of mesoporous spherical TiO_2_. There was no obvious porous character in P25 TiO_2_, as shown the SEM image (Figure S2) and the corresponding pore size distribution curve (Figure 4).

### 2.5. Adsorption Activities

**Figure 5:**
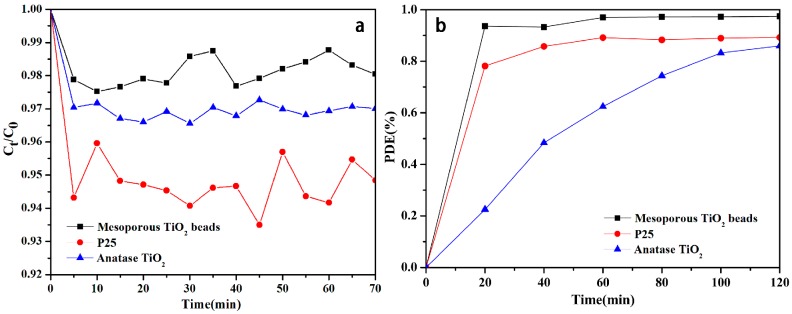
Adsorption and photocatalysis of methylene blue (MB) by mesoporous spherical TiO_2_ and P25 TiO_2_ (C_0_ is the initial concentration of the MB solution, and C_t_ is the concentration of that at different time intervals during the adsorption).

The adsorption activity of mesoporous spherical TiO_2_, P25 TiO_2_, and anatase TiO_2_ (reference material) were demonstrated using methylene blue (MB). As shown in Figure 5a, adsorption occurs when mesoporous spherical TiO_2_, P25 TiO_2_, and anatase TiO_2_ are added to the MB solution. As is shown in Figure 5a, the characteristic absorption peak of MB (~664 nm) decreases dramatically over the first 5 min. This is attributed to the strong electrostatic interaction between positively charged MB and negatively charged TiO_2_ (the existence of OH^−^ radicals by water dissociation can indeed cause dramatic changes to the charge properties of TiO_2_ surface) [22]. With the progress of time, the driving force for adsorption reduces due to decrease in active adsorption sites, and increased repulsion between adsorbed and free MB molecules [23]. The very weak adsorption activity (max ~ 2%) of the mesoporous spherical TiO_2_ when compared to P25 TiO_2_ (max ~ 6%) and anatase TiO_2_ (max ~ 3.5%) makes the current sample very viable from the point of view of recovery and reuse.

### 2.6. Photocatalytic Activities

To evaluate their photocatalytic activities, we induced the photocatalytic decomposition of methylene blue (MB) using irradiation from a 300 W Xe lamp (UV-Light Luminous Instensity: 37 mw/cm^2^). Figure 5b shows the photodegradation efficiency (PDE = (C_0_ − C_t_)/C_0_ × 100%) of MB versus irradiation time. Here C_0_ and C_t_ stand for the concentration of MB at the beginning and after time t. These quantities were estimated by examining the variation of the maximum characteristic adsorption peak intensity of MB. Clearly, mesoporous spherical TiO_2_ shows higher efficiencies (99%) in the photocatalytic degradation of MB than the corresponding P25 TiO_2_ (89%) and anatase TiO_2_ (85%) (for t = 120 min). The photocatalytic oxidation of MB on mesoporous spherical TiO_2_ and P25 TiO_2_ were fitted using pseudo-first order kinetic rate equation. The rate constant (k) for mesoporous spherical TiO_2_ is 0.024 min^−1^, which is much higher than the rate of 0.0066 min^−1^ of P25 TiO_2_ and 0.013 min^−1^ of anatase TiO_2_ [24]. The encouraging photocatalytic kinetics (~364% higher than P25) observed is most likely due to the high surface area and abundant mesoporous structure of mesoporous spherical TiO_2_, which provides channels for the easy diffusion of the MB molecules; this in turn ensures contact between the dye molecule and the photoactive sites [25]. The Tauc plot method (Figure S3) was used to determine the optical band gaps (Eg) of all photocatalysts synthesized. It was found that the band gap was always around 3.3–3.4 eV. Given that the TiO_2_ synthesized here is undoped, this is entirely expected.

### 2.7. The Kinds of Generated ROS under UV-Light Irradiation

**Figure 6:**
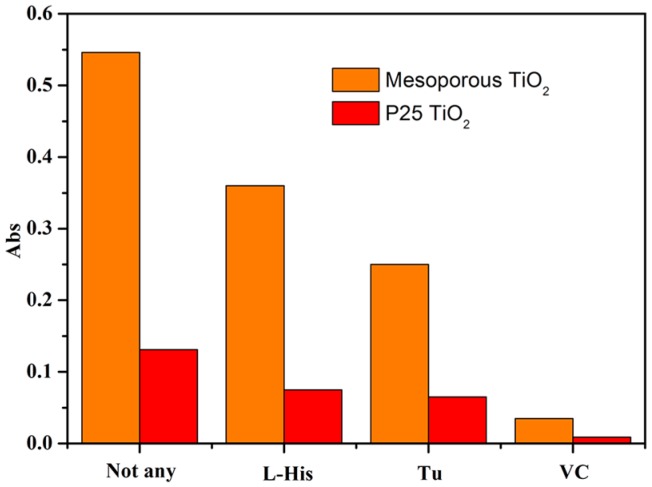
Absorbance of DPCO in DPCI solution in the presence of mesoporous spherical TiO_2_ and P25 TiO_2_ under UV-light irradiation with various quenching reagents. ([DPCI] = 1.00 × 10^−3^ mol/L, [mesoporous spherical TiO_2_] = [P25 TiO_2_] = 1.00 g/L, [L-His] = [VC] = [Tu] = 5.00 × 10^−3^ mol/L, T = 298 K and t = 60 min).

To understand the mechanism of photocatalytic degradation reactions and to determine the kinds of generated ROS, here, several radical scavengers were employed. In general, L-Histidine (L-His) can quench singlet molecular oxygen (^1^O_2_), and Thiourea (TU) can quench the hydroxyl radicals (·OH). Furthermore, Vitamin C (VC) can quench almost all kinds of ROS [26]. During the experiment process, 1,5-diphenyl carbonzide (DPCI) was used as capture agent. It could be oxidized by generated ROS into 1,5-diphenyl carbonzone (DPCO), showing a strong absorbance at 563 nm. Based on the quenching results from different radical scavengers, the kinds of ROS can be determined. For UV-light irradiation, in the absence of any radical scavenger, the higher absorbance of DPCO can be found in Figure 6. This indicates that many ROS generate and then the most DPCI molecules are oxidized to DPCO. The order is mesoporous spherical TiO_2_ > P25 TiO_2_. Apparently, under UV-light irradiation the mesoporous spherical TiO_2_ can generate relatively more ROS than P25 TiO_2_. After the addition of scavengers, the absorption peaks of DPCO obviously weaken for two courses. After the addition of L-His, the absorbance (mesoporous spherical TiO_2_ sample) is obviously higher than that after the addition of TU. The reason for this is that mesoporous spherical TiO_2_ mainly produces ·OH, and, similarly, P25 TiO_2_ generates both ^1^O_2_ and ·OH.

The mechanism of photocatalytic reaction is shown in Figure S4. Under the excitation of UV-light, some electrons are transited from the valence band (VB) of semiconductors to the conduction band (CB). At the same time, electron-hole pairs are formed. These electrons and holes react with the molecular oxygen (O_2_) and water molecules (H_2_O) absorbed on the surface of TiO_2_, respectively. This produces superoxygen radical anions (O_2_^−^), hydroxyl radicals (OH), and singlet oxygen (^1^O_2_), all of which have strong oxidation abilities [27,28]. Mesoporous spherical TiO_2_ has a higher specific surface area and crystallinity, which are the most likely reasons for its superiority over anatase TiO_2_ and P25. The average crystal size of the mesoporous spherical TiO_2_ with internal pores is 46 nm, and their pore dominant size is 5 nm (as discussed above). Thus, the average wall thickness of mesoporous spherical TiO_2_ is lower than the characteristic dimension of the reference samples. Therefore, it ensures the efficient migration of the photoexcited electrons and holes to the surface, and results in the lower recombination probability that is responsible for the observed photoactivity. 

## 3. Experimental Section

### 3.1. Chemicals and Materials

All chemicals were analytical reagents and were used without further purification. The hexadecylamine (HDA; 90%), titanium (IV) isopropoxide (TIP; 98%), absolute ethanol, deionized water, potassium chloride (AR), Degussa P25 TiO_2_, and anatase TiO_2_ were supplied by Sigma-Aldrich, China. Methylene blue (AR grade, Sinopharm, China) was used as a model organic pollutant to evaluate the activity of mesoporous spherical TiO_2_, P25 TiO_2_, and anatase TiO_2_. Benzene, carbon tetrachloride (A.R. grade), 1,5-Diphenyl carbazide (DPCI, 99.0%), L-Histidine (L-His, 99.0%), Thiourea (TU, 99.0%), and Vitamin C (VC, 99.0%) were purchased from Aladdin (China). In all experiments, doubly distilled water was used.

### 3.2. Preparation of Mesoporous Spherical TiO_2_

In a typical process, certain amounts of HDA, HCl, and deionized water were dissolved in 200 mL of anhydrous ethanol. While the above mixture was being continuously stirred for 2 h at room temperature, 4.5 mL of TIP was slowly added, while continuing the stirring. After 2 min, the milky white precursor bead suspension was kept static for 18 h and then washed with ethanol three times, then dried in air at room temperature. After that, the precursor bead samples were transferred into a Teflon-lined autoclave and heated at 160 °C for 16 h by hydrothermal treatment. The resulting precipitate was collected by washing with ethanol and centrifugal separation, and dried in air. Finally, the obtained powders were calcined at 500 °C for 2 h, leading to the formation of mesoporous spherical TiO_2_.

### 3.3. Characterization of TiO_2_

The morphology and particle size of the mesoporous spherical TiO_2_, P25 TiO_2_, and anatase TiO_2_ were examined using a scanning electron microscope (SEM) (JSM-7800F, JEOL, Tokyo, Japan). The phase purity and crystal structure of the obtained samples were examined by X-ray diffraction (XRD) conducted on a PANalytical X’pert diffractometer (Cu Kα, 40 kV, 40 mA). Surface area measurements were performed by nitrogen adsorption using the Brunau–Emmet–Teller (BET) area method on an Accelerated Surface Area and Porosimetry System (ASAP 2420) to obtain the value of specific surface area, pore volume, and mean pore size. 

### 3.4. Adsorption and Photocatalytic Characterization

The adsorption performance of the as-prepared mesoporous spherical TiO_2_ was evaluated via the adsorptive separation of Methylene blue (MB) in aqueous solution, by comparing with commercially available P25 TiO_2_ and anatase TiO_2_ beads. All the adsorption experiments were conducted under stirring conditions throughout the test at room temperature in the dark. The general experimental process was conducted as follows: first, 0.12 g of adsorbent (mesoporous spherical TiO_2_, P25 TiO_2_, or anatase TiO_2_) was added to 200 mL of MB solution with an initial concentration of 25 mg/L, followed by stirring. At 5-min time intervals, the aliquots were withdrawn from the suspension and the powders were separated from the suspension via centrifugation at 8000 rpm. The concentration of residual MB in the supernatant solution was detected using a UV-visible spectrophotometer (Hitachi U-3900, Tokyo, Japan) and was calculated from the maximum peak using a standard calibration curve.

The evaluation of the photocatalytic activity of the samples for the photocatalytic removal of MB in aqueous solution was performed at ambient temperature. The reaction suspension was prepared by adding 0.12 g of photocatalyst powders (mesoporous spherical TiO_2_, P25 TiO_2_, or anatase TiO_2_) into 200 mL of MB solution. Before irradiation, the suspensions were sonicated for 3 min and then magnetically stirred under dark conditions for 30 min to establish adsorption/desorption equilibrium. The suspension was then irradiated under UV light (300 W Microsolar 300 UV—Xe lamp with the wavelength of 10–420 nm, Perfect light). After 20-min irradiation intervals, a 3.0-mL sample was taken from the reaction suspension and centrifuged to remove the photocatalyst powders for analysis. The concentration of MB was determined by the maximum absorption peaks. All the photocatalytic measurements mentioned were repeated twice to ensure the reliability of the results. 

### 3.5. Evaluation of Reactive Oxygen Species (ROS)

To detect the kinds of reactive oxygen species (ROS) generated during pthe hotocatalysis process, three scavengers were used to quench the different ROS. Firstly, 10.00 mL DPCI stock solutions (1.00 × 10^−2^ mol/L) were added into eight 100-mL flasks and marked a-h, respectively. Then, 100 mg mesoporous spherical TiO_2_ and P25 TiO_2_ powders were added to above solutions, respectively. 10 mL L-His, TU, and VC stock solutions (5.00 × 10^−2^ mol·L^−1^) were added into c to h, respectively. All of the eight solutions were diluted to 100 mL with double distilled water. Then, the conical flasks were placed directly under UV-light. The system temperature was controlled at 25.0 ± 0.2 °C. After 1 h, all samples were extracted by the mixed solvent of benzene and carbon tetrachloride (volume ratio = 1:1). They were then diluted to 10.00 mL with the same mixed benzene–carbon tetrachloride solution. The absorbances of all the solutions were determined. 

## 4. Conclusions

In conclusion, we present a simple and efficient method to prepare mesoporous spherical TiO_2_ by soft-template synthesis. The mesoporous spherical TiO_2_ exhibited higher photocatalytic activity than P25 TiO_2_ and anatase TiO_2_. This significant improvement in the photocatalytic performance of mesoporous spherical TiO_2_ arises from the following synergistic effects (i) high surface area; (ii) improved crystallinity; (iii) narrow pore wall thicknesses (ensuring the rapid migration of photogenerated carriers to the surface of the material); and (iv) greater ROS generation under UV-light. Thus, the material reported here has considerable future prospects for photocatalytic and related applications. Modified TiO_2_ can be used as an efficient catalyst for pollutant degradation and organic synthesis under visible light irradiation in the future.

## Supplementary Materials

Supplementary materials are available online.
