# Supplementary material for: Soft-Template Synthesis of Mesoporous Anatase TiO2 Nanospheres and Its Enhanced Photoactivity
Source: Molecules. 2017 Nov 10;22(11):1943. doi: 10.3390/molecules22111943 (PMC6150209; doi:10.3390/molecules22111943)
Supplement: Supplementary file 1 [file molecules-22-01943-s001.pdf]

Supplementary File

Soft-template synthesis of mesoporous anatase TiO_2 nanospheres and its enhanced photoactivity

Xiaojia Li

Results:

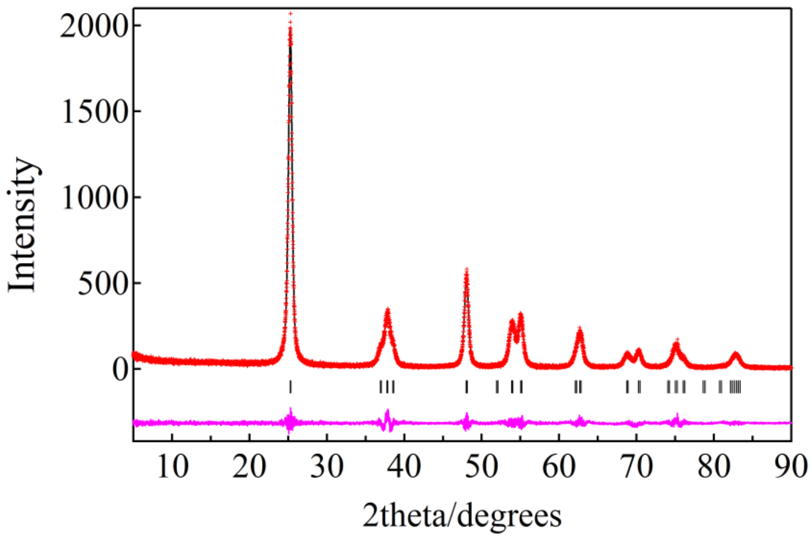

Figure S1. Refinement of PXRD of mesoporous spherical TiO_2.

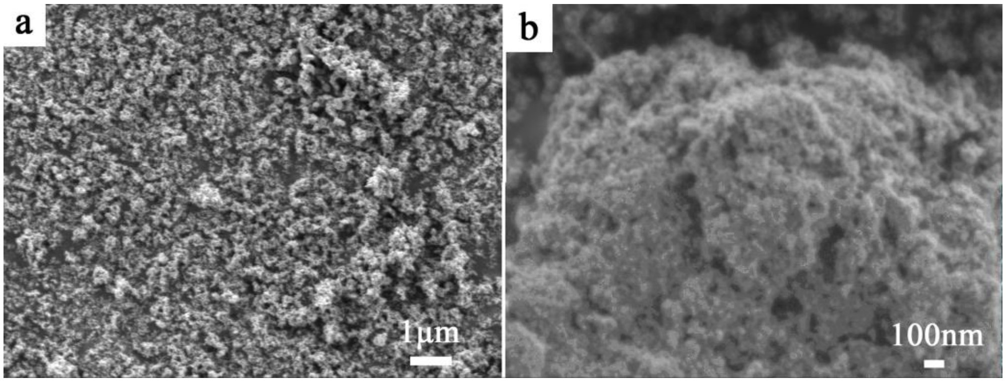

Figure S2. SEM of P25 TiO_2 (a and b).

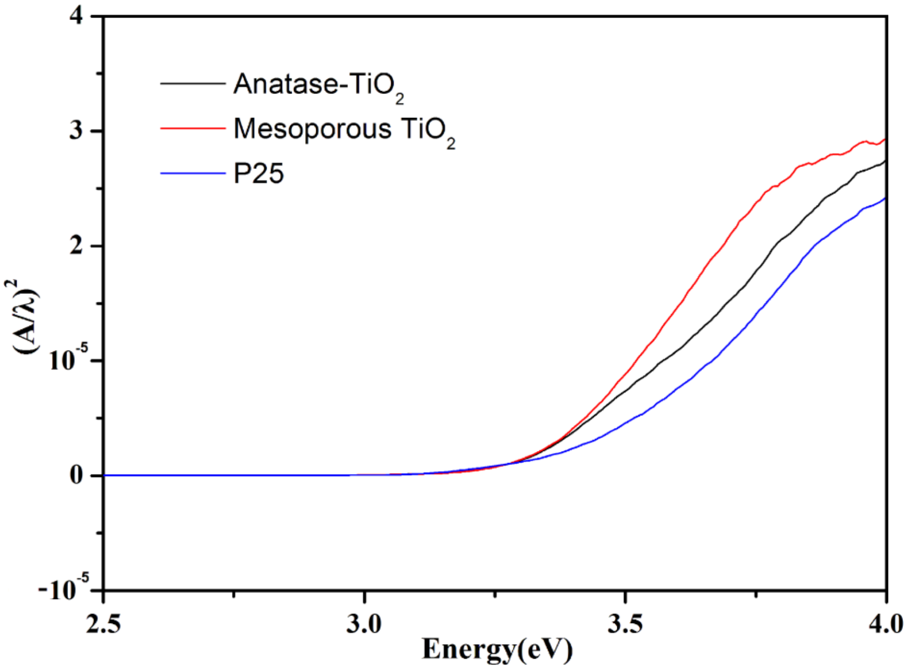

Figure S3. Tauc plot of mesoporous spherical TiO_2, anatase TiO_2 and P25 TiO_2.

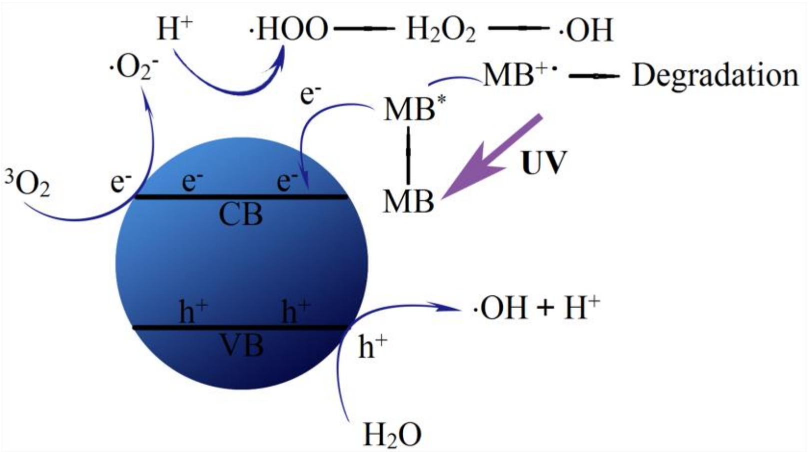

Figure S4. Illustration of photocatalysis degradation of TiO_2 to Methylene blue (MB) under UV-light irradiation.
